# Mixed pain: has pain medicine overlooked Its most common phenotype for over 20 years? A perspective on mechanistic interaction, the nociplastic paradox, and the future of pain classification

**DOI:** 10.3389/fmed.2026.1961489

**Published:** 2026-09-03

**Authors:** Rainer Freynhagen, Antonio Alcántara Montero

**Affiliations:** 1 Department of Anaesthesiology, Critical Care Medicine & Pain Therapy, Benedictus Hospital Feldafing & Tutzing, Feldafing, Germany; 2 Department of Anaesthesiology, Klinikum Rechts der Isar, Technische Universität München, Munich, Germany; 3 Centro de Salud Trujillo, Trujillo, Cáceres, Spain

**Keywords:** central sensitization, mechanistic interaction, mixed pain, neuropathic pain, nociceptive pain, nociplastic pain, pain classification, pain phenotyping

## Abstract

Pain medicine has progressively evolved from a binary classification of nociceptive and neuropathic pain toward a broader mechanistic framework that now includes nociplastic pain. While this evolution has substantially improved our understanding of chronic pain, it has not resolved a persistent clinical reality: most patients do not present with mechanistically pure pain states. Instead, nociceptive, neuropathic, and nociplastic mechanisms frequently coexist within the same individual and interact in ways that challenge traditional classifications. The concept of mixed pain emerged more than two decades ago to capture this complexity. Despite growing recognition across conditions such as back pain, osteoarthritis, cancer pain, postsurgical pain, and fibromyalgia, mixed pain remains largely absent from formal pain taxonomy. At the same time, the increasing acceptance of nociplastic pain has revealed an important paradox. Current terminology explicitly acknowledges the coexistence of nociceptive and nociplastic mechanisms but provides no equivalent conceptual framework for neuropathic–nociplastic overlap, despite growing evidence that this combination is common and clinically relevant. Mixed pain should not be viewed as a diagnosis, a substitute for mechanistic assessment, or a label for pain of unknown origin. Rather, it requires the identification of at least two clinically relevant pain mechanisms contributing to the same clinical presentation. According to the recently accepted international consensus definition, mixed pain is pain associated with a lesion, disease, or disorder resulting in an overlap of at least two mechanistic descriptors (nociceptive, neuropathic, or nociplastic). Thus, mixed pain provides a framework for mechanistic integration rather than mechanistic replacement, helping to prevent its misuse as a diagnostic shortcut. Beyond simple coexistence, emerging evidence suggests that interaction between pain mechanisms may itself have biological and clinical significance. Mixed pain may influence symptom burden, treatment response, and prognosis in ways that cannot be fully explained by individual mechanisms alone. The next frontier in pain medicine may therefore not be the discovery of additional pain mechanisms, but a better understanding of how established mechanisms interact. Twenty years after the first challenge to mechanistic purity, the question is no longer whether pain mechanisms coexist. The question is whether we have underestimated the importance of their interaction.

## Introduction

### The phenotype we keep seeing but rarely classify

For decades, pain medicine has pursued mechanistic precision. The field has progressively evolved from symptom-based descriptions toward mechanism-based classification, distinguishing nociceptive pain from neuropathic pain and, more recently, recognizing nociplastic pain as a third mechanistic descriptor (1). This evolution has undoubtedly advanced our understanding of chronic pain and has provided a more rational basis for diagnosis, phenotyping, and treatment. Yet despite these advances, an uncomfortable reality remains: most patients encountered in clinical practice do not present with mechanistically pure pain states. Instead, they present with combinations of mechanisms.

This observation is neither controversial nor new. Chronic pain affects nearly one in five adults worldwide and remains among the leading causes of disability, healthcare utilization, and reduced quality of life (2). Across virtually every chronic pain condition, clinicians encounter patients whose symptoms cannot be adequately explained by a single mechanism. Osteoarthritis may combine inflammatory nociceptive drivers with neuropathic descriptors and altered central pain processing (3, 4). Chronic low back pain frequently includes nociceptive, neuropathic, and nociplastic features within the same individual (5). Similar patterns are observed in cancer pain, persistent postsurgical pain, complex regional pain syndrome (CRPS), spinal cord injury pain, chemotherapy-induced neuropathy, and numerous musculoskeletal disorders (6–8). The coexistence of mechanisms is therefore not the exception; it may be the rule. This reinforces the idea that mechanistic overlap is a fundamental characteristic of chronic pain rather than a marginal phenomenon.

Indeed, Toda provocatively argued that truly pure nociceptive pain may itself be rare in routine clinical practice (9). While intentionally provocative, this statement highlights an important tension within contemporary pain medicine. If mechanistic overlap is common across chronic pain conditions, why does pain classification continue to behave as though mechanistic purity were the norm? Part of the answer may be historical.

Classification systems are designed to simplify complexity. They provide structure, facilitate communication, support research, and guide treatment decisions. Mechanistically pure categories are easier to define, easier to study, and easier to operationalize than interacting biological systems. Yet simplicity comes at a cost. When classifications prioritize separation, they risk underestimating interaction. This conceptual gap becomes increasingly evident as clinical presentations grow more heterogeneous and mechanistically layered.

This tension has become increasingly apparent with the emergence of nociplastic pain. The recognition that altered nociception and central pain processing can contribute substantially to symptom generation represented a major conceptual advance (1, 10–12). Importantly, nociplastic pain was not introduced because clinicians suddenly encountered a new disease. It was introduced because existing classifications could no longer adequately explain what clinicians were already seeing. In many ways, mixed pain emerged from the same observation.

Both concepts reflect the realization that pain rarely conforms to mechanistic purity. The difference is that nociplastic pain became incorporated into formal terminology, whereas mixed pain remained largely a clinical construct. This discrepancy is difficult to ignore.

Over the last two decades, mixed pain has repeatedly appeared across diseases, specialties, and healthcare systems. International recommendations now recognize mixed pain as highly prevalent, clinically burdensome, and frequently underrecognized (7). Importantly, arguing for mixed pain does not mean arguing against nociceptive, neuropathic, or nociplastic pain. These descriptors remain essential. They have transformed pain medicine and continue to provide the foundation for mechanism-based assessment. The issue is not whether these mechanisms exist. The issue is whether their interaction matters. Current classifications are highly effective at identifying individual mechanisms. They are considerably less effective at describing what happens when those mechanisms coexist, interact, amplify one another, and evolve over time. Yet these interactions may be precisely what clinicians encounter most frequently. Recognizing this gap is essential for aligning pain taxonomy with real-world clinical complexity.

This perspective therefore begins with a simple proposition: perhaps mixed pain is not a marginal concept attempting to challenge established classifications. Perhaps it reflects the dominant clinical phenotype that classifications have struggled to capture. If so, the question is no longer whether pain mechanisms coexist—clinical practice answered that long ago. The more important question is whether pain medicine has underestimated the importance of their interaction. This gap has become even more evident now that an international consensus definition of mixed pain has been established, providing a clearer conceptual anchor for a phenomenon clinicians have recognized for decades.

## Mixed pain came first

Mixed pain did not emerge because pain became more complex. It emerged because our classifications were too simple. The concept of mixed pain was first introduced in 2004 by the German Research Network on Neuropathic Pain (DFNS), proposing that pain may arise from more than one mechanism and challenging the prevailing assumption that chronic pain conditions could be adequately explained by a single mechanistic descriptor (13). Over the following two decades, the concept of mixed pain continued to reappear across diseases, specialties, and countries. Despite differences in terminology, the message remained remarkably consistent: many patients simply did not fit mechanistically pure categories. In this sense, mixed pain came first. It emerged long before nociplastic pain entered formal terminology and reflected a clinical reality that pain taxonomy was not yet prepared to accommodate.

The subsequent development of pain taxonomy followed a different but related path. In 2008, the International Association for the Study of Pain (IASP) revised the definition of neuropathic pain, establishing a more precise mechanistic framework based on a lesion or disease of the somatosensory nervous system. In 2017, IASP introduced nociplastic pain as a third mechanistic descriptor, acknowledging that altered nociception may contribute to pain even in the absence of clear evidence of tissue damage or a somatosensory system lesion or disease (14).

However, these important taxonomic advances did not resolve the original problem that had motivated the mixed pain concept: many patients cannot be adequately explained by one mechanism alone. Rather than replacing the mixed pain concept, the recognition of nociplastic pain made it even more relevant, because it added another clinically important mechanistic descriptor that may coexist and interact with nociceptive and neuropathic drivers. This historical sequence underscores that mixed pain was not created to fill a conceptual gap, but to describe a clinical reality that predated later taxonomic refinements.

A major milestone followed in 2019 with the publication of the first comprehensive international narrative review on mixed pain (6). Beyond summarizing the available evidence, this review proposed the first conceptual definition of mixed pain and highlighted its clinical relevance as a condition arising from the coexistence and interaction of different pain mechanisms within the same patient. Importantly, the concept was never intended to be restricted to a simple overlap between nociceptive and neuropathic pain. By the time of publication, nociplastic pain had already been introduced by the IASP, and the review explicitly acknowledged the contribution of altered central pain processing within mixed clinical presentations. In retrospect, the principal limitation of the original concept may not have been its scope, but its complexity. While clinically intuitive, it lacked a sufficiently simple and operational definition. As a result, mixed pain was often reduced to a nociceptive–neuropathic overlap despite the broader mechanistic perspective originally envisioned. This simplification likely contributed to underrecognition of mixed pain in settings where nociplastic features were present but not explicitly considered.

However, building on this foundation, the first international clinical recommendations for the assessment and management of mixed pain were published in 2025, while bibliometric analyses demonstrated a marked increase in scientific interest in the field (7, 15).

Finally, an international Delphi consensus process resulted 2026 in the first internationally accepted consensus definition of mixed pain, marking the transition from an intuitive clinical concept to a structured international framework. This definition formally establishes mixed pain as pain that is associated with a lesion, disease, or disorder resulting in an overlap of at least two mechanistic pain descriptors (nociceptive, neuropathic, or nociplastic) (16). This consensus provides the conceptual clarity that had been missing for more than two decades and offers a stable foundation for future clinical and research applications.

**Figure 1:**
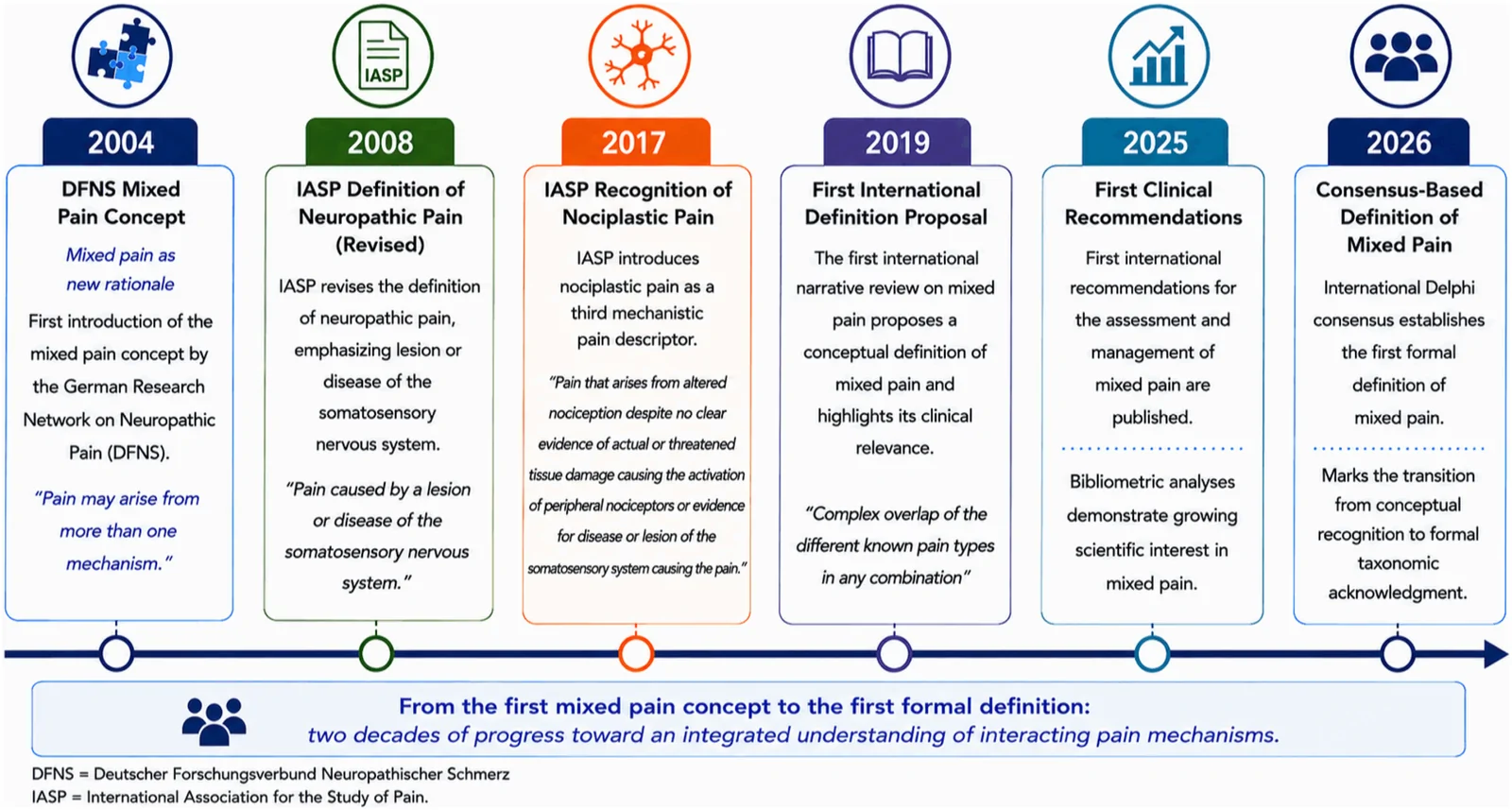
Evolution of the mixed pain concept and related pain taxonomy. Key milestones in the evolution of the mixed pain concept from its original description by the German Research Network on Neuropathic Pain (DFNS) in 2004 to the recent consensus-based definition of mixed pain. The timeline highlights major developments in pain taxonomy, including the revised IASP definition of neuropathic pain (2008), recognition of nociplastic pain (2017), publication of the first conceptual definition of mixed pain within a narrative review (2019), and the first international clinical recommendations for mixed pain (2025) culminating in the first international consensus definition of mixed pain (2026).

Seen from today's perspective, the history of mixed pain is not the story of a concept that expanded over time. It is the story of a concept that is gradually being simplified, clarified, and brought closer to what clinicians have been observing all along (Figure 1). Perhaps that is why mixed pain has refused to disappear. And perhaps that is why an uncomfortable question remains: If clinicians have been observing the coexistence of pain mechanisms for more than twenty years, why has pain classification remained so committed to mechanistic separation?.

## The nociplastic paradox

Few concepts have influenced modern pain medicine as profoundly as nociplastic pain. Its introduction by the IASP filled an important conceptual gap and acknowledged what clinicians had long suspected: altered pain processing can become a major driver of chronic pain, even when tissue pathology or somatosensory injury alone cannot fully explain the patient's symptoms (1). Yet the success of the concept has revealed an important paradox. According to current IASP terminology, patients may have a combination of nociceptive and nociplastic pain. This coexistence is explicitly acknowledged in the explanatory note accompanying the definition of nociplastic pain (14). However, no equivalent statement exists for neuropathic and nociplastic pain. This asymmetry is difficult to ignore as it creates a conceptual imbalance that does not reflect everyday clinical reality.

Clinicians routinely encounter patients with painful diabetic neuropathy, postherpetic neuralgia, chemotherapy-induced peripheral neuropathy, spinal cord injury pain, CRPS, or chronic radiculopathy who simultaneously exhibit widespread hypersensitivity, disproportionate pain intensity, fatigue, sleep disturbance, cognitive complaints, and widespread pain extending beyond the territory of the original lesion. In other words, they encounter patients with neuropathic and nociplastic features every day. The inconsistency becomes even more difficult to reconcile when considering fibromyalgia. Traditionally regarded as the prototypical nociplastic pain condition, emerging evidence suggests that many patients also display clinically relevant nociceptive and neuropathic contributors (17). The paradox is therefore not that mechanisms overlap. The paradox is that current terminology explicitly recognizes nociceptive–nociplastic coexistence (5, 12, 14, 17, 18) while remaining largely silent regarding neuropathic–nociplastic coexistence.

From a biological perspective, this distinction appears increasingly difficult to defend. If altered central pain processing can amplify nociceptive input, why should it not amplify neuropathic input? Why should central sensitization selectively interact with inflammation, tissue injury, or mechanical loading, but not with nerve injury, ectopic activity, or deafferentation? Current neurobiology offers little support for such a separation (10, 19, 20). Indeed, contemporary concepts of central sensitization suggest precisely the opposite: central amplification is fundamentally a modulatory process. It acts upon incoming signals regardless of whether their origin is nociceptive or neuropathic. If this interpretation is correct, coexistence of neuropathic and nociplastic mechanisms should not be viewed as exceptional. It should be expected.

Importantly, this criticism is not directed against the concept of nociplastic pain itself. The introduction of nociplastic pain was both necessary and transformative. The issue is not that the framework is wrong. The issue may be that it remains incomplete. Mixed pain offers one possible solution. Rather than forcing clinicians to choose between competing descriptors, it acknowledges that nociceptive, neuropathic, and nociplastic mechanisms may coexist within the same patient and that their interaction may be clinically relevant. The central question is therefore no longer whether individual mechanisms exist. The central question may be whether current classifications have underestimated the importance of their interaction. Addressing this gap may help align mechanistic terminology with real-world clinical complexity.

## Mixed pain is not a diagnosis

One of the most common criticisms of mixed pain is that it risks becoming a diagnostic shortcut. This concern is understandable. Whenever a new clinical construct is introduced, there is a danger that it becomes a convenient label applied whenever patients fail to fit existing classifications. In such a scenario, mixed pain could quickly evolve into a repository for uncertainty rather than a framework for understanding. If that were the case, the criticism would be justified. However, this interpretation fundamentally misunderstands the concept. Mixed pain was never intended to replace mechanistic assessment. It was intended to depend on it.

A patient cannot be classified as having mixed pain simply because the pain is complex. A patient cannot be classified as having mixed pain because treatment has failed. A patient cannot be classified as having mixed pain because the clinician is uncertain. And mixed pain should never become a synonym for “pain of unknown origin.” On the contrary, mixed pain becomes meaningful only after identifiable mechanisms have been established.

Recent international consensus efforts have therefore focused on clarifying a principle that has occasionally been overlooked: mixed pain is not a diagnosis in itself, but a descriptor of mechanistic coexistence. At its core, mixed pain requires the coexistence of at least two clinically relevant pain mechanisms contributing to the same clinical presentation, a principle now formally incorporated into the accepted international consensus definition (16). This distinction is more than semantic. Without identifiable mechanisms, mixed pain becomes meaningless. With identifiable mechanisms, it becomes clinically useful.

Indeed, one could argue that mixed pain requires greater diagnostic rigor rather than less. The clinician must actively assess nociceptive drivers, neuropathic features, and manifestations of altered central pain processing before concluding that multiple mechanisms contribute to the patient's pain experience. The objective is not to identify a single dominant mechanism. The objective is to understand the complete mechanistic landscape. It reinforces the need for structured mechanistic assessment rather than intuitive classification.

This shift has important clinical consequences. Many treatment failures in chronic pain may not arise because available therapies are ineffective, but because mechanistic assessment remains incomplete. Clinicians frequently target the most obvious mechanism while overlooking others that continue to drive symptoms. Nonsteroidal anti-inflammatory drugs may fail because neuropathic contributors remain unrecognized. Neuropathic pain medications may fail because ongoing nociceptive drivers continue to dominate the clinical presentation. Surgical interventions may fail because central amplification mechanisms remain unaddressed. When treatment fails, the response is often escalation rather than reassessment. Recognizing mixed mechanistic contributions may therefore prevent unnecessary treatment escalation and improve therapeutic alignment.

Mixed pain encourages a different form of reasoning. Instead of asking: What is the mechanism responsible for this patient's pain? clinicians may increasingly need to ask: Which mechanisms contribute to this patient's pain, and how do they interact? The difference may appear subtle. Its implications are not.

Perhaps this is where the true value of mixed pain resides. Not in creating another category, but in encouraging clinicians to move beyond mechanistic reductionism and toward mechanistic integration. Importantly, this perspective aligns with the original intent of the mixed pain concept. Mixed pain was never conceived as an alternative to diagnosis. It was conceived as a way of describing a clinical reality that conventional classifications struggled to capture. In this sense, mixed pain serves as a bridge between mechanistic precision and clinical complexity.

Perhaps the most important unresolved question surrounding mixed pain is whether it simply describes coexistence—or whether interaction between mechanisms creates something qualitatively different. For years, mixed pain has largely been viewed as an overlap construct. Under this interpretation, a patient with nociceptive and neuropathic pain simply has nociceptive pain plus neuropathic pain. The concept remains descriptive but adds little beyond acknowledging coexistence. This interpretation is reasonable, but it may also be incomplete.

Biological systems rarely behave as the simple sum of their individual components. Interactions between mechanisms often generate properties that cannot be predicted by examining each process in isolation. Immunology, oncology, and neuroscience provide countless examples in which interaction becomes as important as the individual mechanisms themselves. Why should pain be different?.

Clinical observations increasingly suggest that patients with mixed pain differ from those with more mechanistically homogeneous presentations. Across multiple conditions, patients with mixed pain frequently exhibit a greater symptom burden, higher levels of disability, increased healthcare utilization, more psychological comorbidity, longer diagnostic delays, higher opioid consumption, more sleep disturbance, and less predictable treatment responses than patients with seemingly purer pain phenotypes, with some studies reporting diagnostic delays approaching three years and opioid use rates nearly threefold higher than those observed in more mechanistically homogeneous pain populations (7, 21–25). Importantly, these observations often appear disproportionate to what would be expected from individual mechanisms alone. This raises an intriguing possibility: what if interaction itself contributes to symptom expression? What if the coexistence of nociceptive, neuropathic, and nociplastic mechanisms creates emergent properties that cannot be fully explained by any mechanism individually? This hypothesis may help explain the disproportionate clinical burden observed in mixed pain populations.

And several observations support such a hypothesis. First, treatment response in mixed pain often appears less predictable than in mechanistically purer conditions. Clinicians frequently encounter patients who improve only partially when a single mechanism is targeted, yet respond more favorably when multiple mechanisms are addressed simultaneously. This observation is one of the reasons why multimodal treatment strategies have become central to modern pain management (7, 26, 27).

Second, symptom profiles in mixed pain often appear qualitatively different rather than merely quantitatively more severe. Patients commonly report combinations of mechanical pain, burning sensations, allodynia, diffuse hypersensitivity, fatigue, sleep disturbances, cognitive dysfunction, emotional distress, and fluctuating symptom patterns. Such presentations can be difficult to reconcile with any single mechanism alone (3–7, 17, 20, 28–30).

Third, emerging clinical evidence increasingly treats mixed pain not merely as a theoretical construct but as a clinically observable phenomenon. Post-mastectomy pain syndrome, for example, has recently been proposed as a model condition in which nociceptive, neuropathic, and central mechanisms coexist and interact within the same patient, providing a real-world illustration of the complexity captured by the mixed pain construct (31). Even more intriguing are emerging physiological and biosignal-based approaches. Recent studies suggest that complex pain phenotypes may exhibit measurable physiological signatures that are not fully explained by individual pain descriptors alone (8).

While these findings remain preliminary, they challenge the assumption that mixed pain simply reflects additive symptom burden. If future studies confirm reproducible physiological signatures associated with mixed pain states, the concept may move beyond a descriptive framework and become a biologically testable hypothesis.

Perhaps most importantly, the hypothesis aligns with contemporary views of pain as a dynamic network phenomenon. Nociceptive input may facilitate central sensitization. Neuropathic injury may amplify inflammatory signaling. Central amplification may increase the impact of both nociceptive and neuropathic inputs. Psychological, behavioral, and contextual factors may further modify each of these interactions. The resulting clinical presentation emerges not from a single mechanism, but from the interaction of multiple mechanisms operating simultaneously (4).

From this perspective, mixed pain may be better understood as a system rather than a category. Importantly, this does not mean that mixed pain should already be considered a distinct mechanistic descriptor. Current evidence remains insufficient to support such a conclusion. No validated biomarker exists. Diagnostic criteria continue to evolve. Mechanistic heterogeneity remains substantial. Yet it may be equally premature to dismiss the possibility. Future research may determine whether mixed pain represents not only coexistence but a distinct interaction-driven phenotype.

Historically, major advances in pain classification often began with clinical observations that challenged existing frameworks. Neuropathic pain emerged because tissue injury alone could not explain certain symptom patterns. Nociplastic pain emerged because nociceptive and neuropathic mechanisms alone could not adequately explain many chronic pain presentations. Mixed pain may represent a similar challenge. The debate is not whether pain mechanisms coexist—that reality has been evident in clinical practice for decades—but whether their interaction creates a distinct biological and clinical phenotype. If so, mixed pain may be more than a descriptive construct. We are not proposing a fourth pain descriptor. Instead, we are asking whether a taxonomy built around isolated mechanisms is sufficient to explain the complexity of chronic pain as it presents in real-world patients.

## A pragmatic clinical framework

Conceptual debates are important. Clinical decisions are unavoidable. Most patients with chronic pain are not managed in specialized pain centers. They are treated in primary care, neurology, orthopedics, rheumatology, oncology, rehabilitation, and surgical practices where consultation time is limited and advanced diagnostic tools are often unavailable. Any clinically useful mixed pain framework must therefore be simple. It must help clinicians navigate complexity without becoming complex itself. A pragmatic approach can be built around three fundamental questions:

### Question 1: Is there a nociceptive driver?

Examples include inflammation, tissue injury, mechanical loading, degenerative pathology, cancer-related tissue damage, or other identifiable peripheral sources capable of activating nociceptors. The presence of a nociceptive driver does not exclude other mechanisms. Indeed, ongoing nociceptive input may facilitate central sensitization and contribute to the development of more complex pain phenotypes over time. This highlights the importance of reassessing nociceptive contributors even when other mechanisms are present.

### Question 2: Are neuropathic features present?

Examples include burning pain, electric shocks, allodynia, sensory deficits, hyperalgesia, dermatomal or neuroanatomically plausible symptom distribution, or evidence of a lesion or disease affecting the somatosensory nervous system. Importantly, neuropathic pain should not be restricted to classical textbook presentations. Many patients exhibit neuropathic features without displaying a completely “pure” neuropathic phenotype. The identification of neuropathic contributors therefore represents one element of mechanistic assessment rather than the final diagnosis itself. Screening tools such as painDETECT, DN4, and LANSS can be useful adjuncts for identifying neuropathic pain characteristics in routine practice. Although they are not diagnostic, they may facilitate mechanistic phenotyping and help detect clinically relevant neuropathic contributors that might otherwise remain unrecognized (28–30, 32, 33). It is therefore essential to ensure that neuropathic components are not overlooked in mixed pain presentations.

### Question 3: Are there signs of altered central pain processing?

Importantly, nociplastic contributions should not be inferred from pain intensity alone. Increasing pain widespreadness beyond anatomically plausible nociceptive or neuropathic territories may represent one of the strongest clinical clues suggesting altered central pain processing. Additional indicators include widespread hypersensitivity, fatigue, sleep disturbance, cognitive complaints, symptom amplification, and poor response to peripheral interventions. Although none of these features are diagnostic individually, their combination substantially increases the likelihood of a clinically relevant nociplastic component (34). From a practical perspective, clinicians may therefore benefit from looking beyond pain intensity alone.

The purpose of these questions is not to assign a label. The purpose is to identify clinically relevant mechanisms. When two or more domains appear clinically important, mixed pain should be considered. Importantly, this framework does not replace diagnosis. It does not eliminate the need to identify underlying pathology. Nor should mixed pain ever become a synonym for uncertainty. Rather, it provides a pragmatic way of organizing mechanistic reasoning. The framework aligns with recent consensus efforts that emphasize coexistence of multiple clinically relevant pain mechanisms while explicitly distinguishing mixed pain from pain of unknown origin (16). This alignment strengthens the clinical utility of the framework across diverse practice settings.

**Figure 2:**
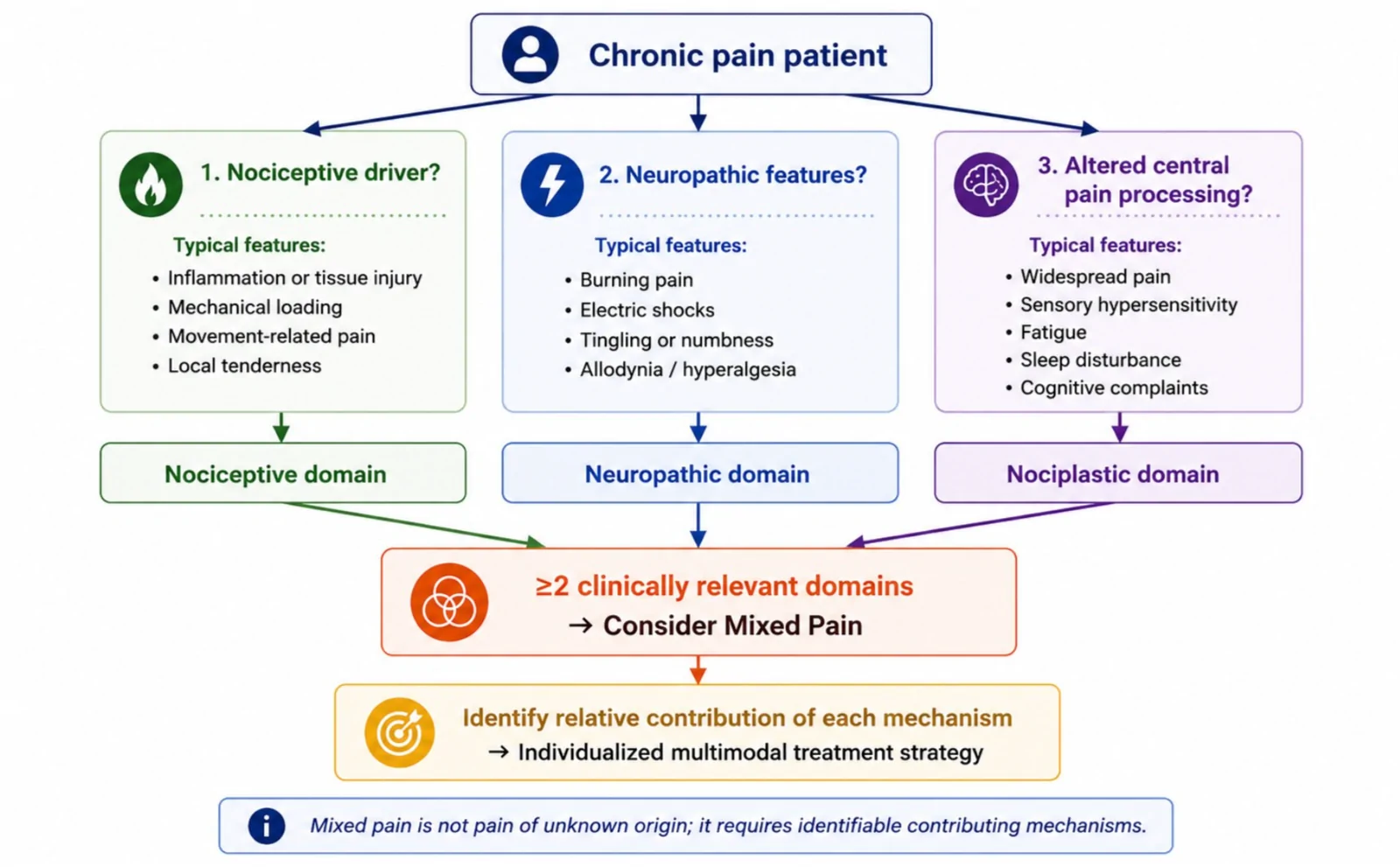
A pragmatic clinical framework for mixed pain. Three-question heuristic for the identification of mixed pain in routine clinical practice: 1. Is there a nociceptive driver? 2. Are neuropathic features present? 3. Are there signs of altered central pain processing?. When two or more domains appear clinically relevant, mixed pain should be considered. The framework is intended to complement, not replace, diagnostic assessment and emphasizes identification of underlying mechanisms rather than pain of unknown origin.

And such a framework is important because a patient with nociceptive and neuropathic contributors may require a different treatment strategy than a patient with either mechanism alone. A patient with neuropathic and nociplastic features may require different expectations regarding treatment response and prognosis. And a patient exhibiting all three domains may require a fundamentally multimodal approach from the outset (27, 35). Mixed pain therefore becomes less a diagnosis and more a way of thinking—a framework that shifts attention away from mechanistic competition and toward mechanistic integration. The practical application of this concept is illustrated in Figure 2.

## Conclusions: the next frontier

For more than three decades, clinicians have encountered patients whose pain could not be adequately explained by mechanistic purity alone. The mixed pain concept emerged from this reality long before the nociplastic pain era, yet it remains largely absent from formal pain taxonomy. Recent international consensus efforts may help move the field beyond terminology and toward a more important question: not which mechanisms are present, but how they interact (16).

The challenge is no longer to identify additional mechanisms. It is to understand the biological and clinical consequences of mechanistic interaction. Mixed pain may ultimately prove to be more than a descriptor—not because it replaces existing mechanistic categories, but because it highlights the limitations of viewing pain through isolated mechanistic lenses. If the coexistence and interaction of nociceptive, neuropathic, and nociplastic mechanisms influence symptom burden, treatment response, and prognosis in ways that cannot be fully explained by any mechanism alone, then mixed pain may represent a critical dimension of chronic pain that current classifications have yet to capture.

Twenty years after the first formal challenge to mechanistic purity, the field may be approaching a turning point.

## Data Availability

The original contributions presented in the study are included in the article/Supplementary Material, further inquiries can be directed to the corresponding author.
